# Publisher Correction: 3D Shape Modeling for Cell Nuclear Morphological Analysis and Classification

**DOI:** 10.1038/s41598-018-33574-w

**Published:** 2018-10-26

**Authors:** Alexandr A. Kalinin, Ari Allyn-Feuer, Alex Ade, Gordon-Victor Fon, Walter Meixner, David Dilworth, Syed S. Husain, Jeffrey R. de Wet, Gerald A. Higgins, Gen Zheng, Amy Creekmore, John W. Wiley, James E. Verdone, Robert W. Veltri, Kenneth J. Pienta, Donald S. Coffey, Brian D. Athey, Ivo D. Dinov

**Affiliations:** 1 Department of Computational Medicine and Bioinformatics, University of Michigan Medical School, Ann Arbor, MI USA; 2 Statistics Online Computational Resource (SOCR), Department of Health Behavior and Biological Sciences, University of Michigan School of Nursing, Ann Arbor, MI USA; 3 Division of Gastroenterology, Department of Internal Medicine, University of Michigan Medical School, Ann Arbor, MI USA; 4 Department of Urology, James Buchanan Brady Urological Institute, Johns Hopkins University School of Medicine, Baltimore, MD USA; 5 Michigan Institute for Data Science (MIDAS), University of Michigan, Ann Arbor, MI USA

Correction to: *Scientific Reports* 10.1038/s41598-018-31924-2, published online 12 September 2018

The original version of this Article contained a typographical error in the spelling of the author Jeffrey R. de Wet, which was incorrectly given as Jeffrey R. de Wett. This has now been corrected in the PDF and HTML versions of the Article.

