# Supplementary material for: 3D Shape Modeling for Cell Nuclear Morphological Analysis and Classification
Source: Sci Rep. 2018 Sep 12;8:13658. doi: 10.1038/s41598-018-31924-2 (PMC6135819; doi:10.1038/s41598-018-31924-2)
Supplement: Supplementary file 1 — Supplementary Information [file 41598_2018_31924_MOESM1_ESM.pdf]

Supplementary Information

3D Shape Modeling for Cell Nuclear Morphological Analysis and Classification

Alexandr A. Kalinin[1,2,§], Ari Allyn-Feuer[1,§], Alex Ade[1], Gordon-Victor Fon[1], Walter Meixner[1], David Dilworth[1], Syed S. Husain[2], Jeffrey R. de Wet[1], Gerald A. Higgins[1], Gen Zheng[3], Amy Creekmore[3], John W. Wiley[3], James E. Verdone[4], Robert W. Veltri[4], Kenneth J. Pienta[4], Donald S. Coffey[4], Brian D. Athey[1,5,*], Ivo D. Dinov[1,2,5,*]

[1]Department of Computational Medicine and Bioinformatics, University of Michigan Medical School, Ann Arbor, MI, USA

[2]Statistics Online Computational Resource (SOCR), Health Behavior and Biological Sciences, University of Michigan School of Nursing, Ann Arbor, MI, USA

[3]Division of Gastroenterology, Department of Internal Medicine, University of Michigan Medical School, Ann Arbor, MI, USA

[4]Michigan Institute for Data Science (MIDAS), University of Michigan, Ann Arbor, MI, USA

[5]Department of Urology, The James Buchanan Brady Urological Institute, Baltimore, MD, USA

[§]These authors contributed equally to this work

[*]Correspondence to statistics@umich.edu

Validation on synthetic data

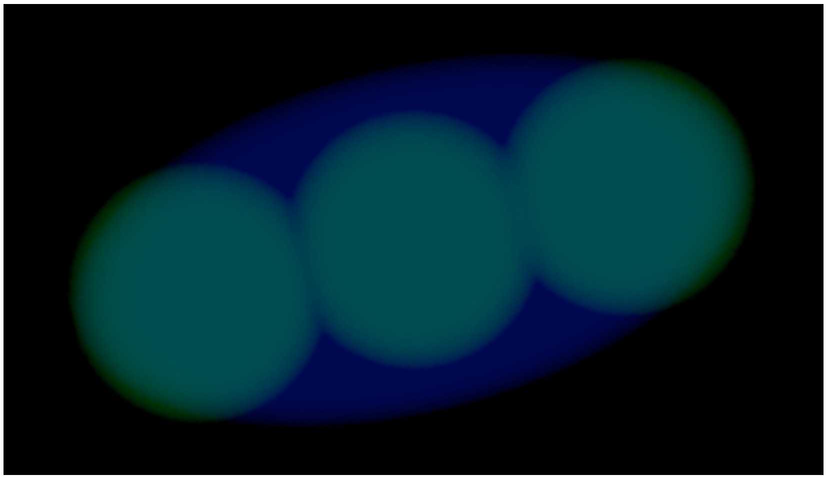

Supplementary Figure S1. Example of synthetic data. Overlapping spheres or ‘beads’ (R=30, overlap=5) within an ellipsoid (a=80, b=40, c=40) used to validate shape morphometry measure calculations and differences in morphometric features.

Supplementary Table S1. Morphometry of synthetic objects. Cube (a=160), octahedron and sphere (R=80)

Primitive	AvgMean Curvature	Compute Area	Volume	Curvedness	Shape Index	Fractal Dimension
**Cube**	0.029	144438.190	4060563.200	0.323	0.012	2.181
**Octahedron**	0.036	42441.207	687556.250	0.349	0.017	2.016
**Sphere**	0.015	79633.500	2111214.800	0.713	0.009	2.095
**Ellipsoid**	0.026	33973.812	526245.700	0.633	0.013	2.097
**Beads**	0.029	29279.334	322649.560	0.612	0.035	2.147

Definitions of morphometric measures

Supplementary Table S2. Core morphometric measures

Geometric Measure	Mathematical Formulas	Details and Interpretation
**Volume**	$V = \iiint_{R^3} I_D(x, y, z)\, dxdydz$	The amount of 3D space enclosed by a closed boundary – inside of a 3D solid – which is quantified numerically in world coordinated (e.g., mm^3 or m^3). The volume of a solid represents the space capacity of the object.
**Surface Area**	$SA = \iint_{\Omega} \lvert \vec{r}_u \times \vec{r}_v \rvert dudv$	The surface area of a 3D solid object is the total area of its (curved) boundary (a 2-manifold). Surface areas of flat polygonal shapes must agree with their geometrically defined area. Volume and Surface area are invariant under the group of Euclidean motions.
**Mean or Gaussian Curvature**	$MC = H = \frac{(\kappa_1 + \kappa_2)}{2}$ $GC = K = \kappa_1 \times \kappa_2$ Example, $GC = -2$ 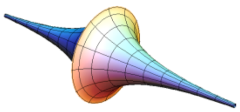 Saddle surface with normal planes in	For an orientable surface defined as a function of two parameters, $z = S(x, y)$, assuming an upward pointing normal, the **mean curvature** is: $MC = -(1/2)\nabla \cdot \left(\frac{\nabla(z-S)}{\lvert\nabla(z-S)\rvert}\right) = (1/2)\nabla \cdot \left(\frac{\nabla S}{\sqrt{1+\lvert\nabla S\rvert^2}}\right) = \left(\frac{1}{2}\right)\frac{\frac{\partial^2 S}{\partial y^2}\left(1+\left(\frac{\partial S}{\partial x}\right)^2\right) - 2\frac{\partial S \partial S}{\partial x \partial y}\frac{\partial^2 S}{\partial x \partial y} + \frac{\partial^2 S}{\partial x^2}\left(1+\left(\frac{\partial S}{\partial y}\right)^2\right)}{\left(1+\left(\frac{\partial S}{\partial x}\right)^2+\left(\frac{\partial S}{\partial y}\right)^2\right)^{3/2}}$. The only surface in R^3 with constant positive mean curvature is the **sphere**. The only surface in R^3 with constant negative Gaussian curvature is the pseudosphere. The Curvature provides (local at each vertex) surface classification: • **Elliptical**: both principal curvatures have the same sign, an

	directions of principal curvatures	d the surface is locally convex.<br>• **Hyperbolic**: the principal curvatures have opposite signs, and the surface will be locally saddle shaped.<br>• **Parabolic**: one of the principal curvatures is zero. Parabolic points generally lie in a curve separating elliptical and hyperbolic regions.
**Shape Index**	$SI = \frac{2}{\pi} arctan\left(\frac{\kappa_2 + \kappa_1}{\kappa_2 - \kappa_1}\right)$	$-1 \leq SI \leq 1$<br>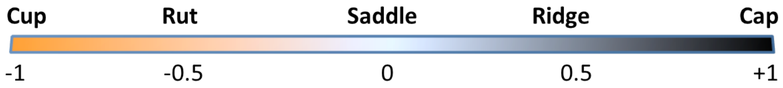
**Curvedness [1]**	$CV=\sqrt{\frac{\kappa_1^2+\kappa_2^2}{2}}$<br>$0 \leq CV$	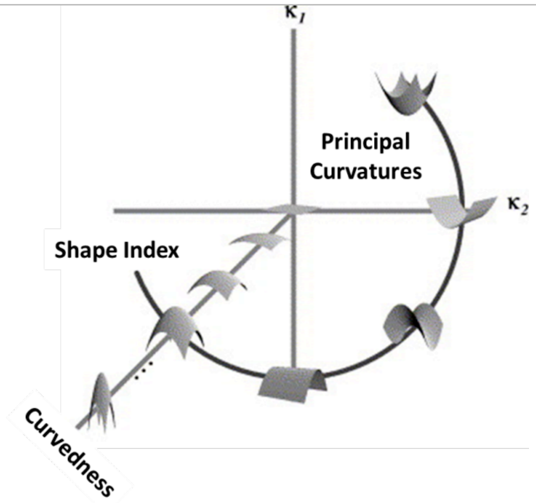
**Fractal Dimension**	$FD = \frac{log(N)}{log\left(\frac{1}{\rho}\right)} = -\frac{log(N)}{log(\rho)}$	Topological dimensions are always integral, however, dimensionality can be extended to all positive real numbers. The fractal dimension can take non-integer values indicating that a set fills its space qualitatively and quantitatively differently than ordinary geometric shapes.

- A curve with fractal dimension=1.10 is similar to an ordinary line, but its 1D length would be infinite.
- A curve with fractal dimension 1.9 would wind through space (R^3) very much like a 2D surface.
- A surface with fractal dimension>2.0 (e.g., of 2.1) would look like a regular 2-manifold (e.g., sphere), but begins to fill R^3 and it's 2D area would be infinite
- A surface with a fractal dimension of 2.9 folds and flows to fill R^3

  like a volume, but it's 3D measure of volume would be trivial (0).
- The Koch curve (below) has a fractal dimension of 1.2619.

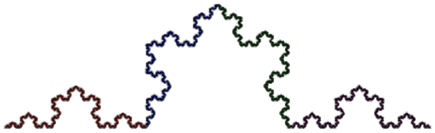

That is, for a fractal described by $N = 4$, $\rho = \frac{1}{3}$, $D = 1.2619$, a non-integer dimension > 1, implying its length is infinite.

Fractal dimension concept is based on self-similarity, scaling and dimension. Classically, measuring the length of a line using first one measuring stick of size 1, then another 1/3 its size, will give for the second stick a total

		length 3 times as many sticks long as with the first. Similarly in 2D – if we measure an area using a square of size 1, and then then measuring the same area a square of size 1/3 we will need 9 times as many squares as with the first measure. To generalize, let N, ρ, and D represent the number of new sticks, the scaling factor, and the fractal dimension, then: $N \cong \rho^{-D} \Rightarrow log_{\rho} N = -D \Rightarrow D = -\frac{logN}{log\rho}$. For the Koch curve, a fractal line is first measured with a stick of size 1, then re-measured using a new stick scaled by 1/3 but requires 4 sticks of this size. In this case, $N = 4$, $\rho = \frac{1}{3}$, and $D = -\frac{logN}{log\rho} = \frac{log4}{log3} = 1.2619.$

Running workflow demo using Pipeline Web Start

This demo is prepared for classification of serum-starved Fibroblast cells (SS, run #160). This workflow take as an input original 16 1024x1024xZ 3D TIFF images (sub-volumes) in DAPI channel (c0) and metadata. It demonstrates nuclear binary mask preparation, 3D shape modeling, morphometric measure extraction, and classification running in distributed mode on a cluster using LONI Pipeline guest mode. It outputs .csv file with image-level output label, nucleus-level accuracy and average probability as well as labels and probabilities for individual nuclear masks

that were segmented out of 3D input sub-volume, passed the curation, 3D shape modeling, feature extraction, and classification.

Instructions below describe how to use Pipeline in a guest mode. If you already have LONI Pipeline credentials you can just download Pipeline Client from http://pipeline.loni.usc.edu/products-services/pipeline-software/ and log in using your username and password.

- Download and install LONI Pipeline Client Web Start from http://pipeline.loni.usc.edu/files/webstart/pipeline.jnlp (requires Java[1])
- Create "Try-It-Now" connection by clicking Connections icon at the bottom-right corner of the client to connect to the server without credentials (enter space for password)
- Download workflow file c0-classification-demo-run160.pipe from http://www.socr.umich.edu/data/3d-cell-morphometry-data/workflows/c0-classification-demo-run160.pipe and open in the Pipeline client
- Click Run button at the bottom of the client – after workflow validates the protocol, presence of input data, and availability of free nodes in cluster, it will start running jobs
- Running the workflow take 2-3 hours on average, depending on availability of computing nodes in the cluster
- After workflow is completed, right-click on Calculate Accuracy module in Classification group and download or view the output file from Output Files tab

[1] If you have problems accessing Java applications using Chrome, Oracle recommends using Internet Explorer (Windows) or Safari (Mac OS X) instead. See https://www.java.com/en/download/faq/chrome.xml.

You can also double-click on group in the workflow at any moment to see individual modules inside. You can disconnect while the workflow is running – under Connections you will be able to see your unique GUEST-ID that you can use to reconnect later and check workflow status (enter space for password). Having your GUEST-ID you should be able to use LONI Pipeline Web App at http://pipeline.loni.usc.edu/webapp/ to reconnect to the same sessions (web app is still in Beta and might not work as expected). Workflow protocol can be ran multiple times to validate reproducibility of the morphometry results. Pipeline documentation, including instructions module definition, modification, and execution, is available on the official website at .

References

1. Norman JF, Todd JT, Norman HF, Clayton AM, McBride TR. Visual discrimination of local surface structure: slant, tilt, and curvedness. Vision Res. 2006;46:1057-1069.
